# Splenectomy Leads to Amelioration of Altered Gut Microbiota and Metabolome in Liver Cirrhosis Patients

**DOI:** 10.3389/fmicb.2018.00963

**Published:** 2018-05-15

**Authors:** Yang Liu, Jun Li, Ye Jin, Lei Zhao, Fuya Zhao, Jing Feng, Aidong Li, Yunwei Wei

**Affiliations:** Department of Oncological and Laparoscopic Surgery, The First Affiliated Hospital of Harbin Medical University, Harbin, China

**Keywords:** liver cirrhosis, splenectomy, microbiota, 16s rRNA gene, metabolome

## Abstract

Dysbiosis of gut microbiota and metabolome is a frequently encountered condition in liver cirrhosis (LC) patients. The severity of liver dysfunction was found to be correlated with the degree of microbial dysbiosis. Several clinical studies have indicated liver function improvement after therapeutic splenectomy for LC-induced hypersplenism. We sought to determine whether such post-splenectomy outcome is pertinent to modulation of the abnormal gut microenvironment in LC patients. A cross-sectional study including 12 LC patients and 16 healthy volunteers was first conducted, then a before–after study in the cohort of patients was carried out before and 6 months after splenectomy. Fecal samples were collected in hospital. Temporal bacterial (*n* = 40) and metabolomics (*n* = 30) profiling was performed using 16s rRNA gene sequencing and ultra performance liquid chromatography/mass spectrometer (UPLC/MS), respectively. Our results revealed that microbial composition in patients was clearly different from that in healthy controls (HCs), evidenced by considerable taxonomic variation. Along with improved liver function (Child–Pugh score), the patients also displayed similar gut microbiota profile and predicted metagenome function to that of HCs after splenectomy. *Enterobacteriaceae* and *Streptococcaceae*, two LC-enriched families showing positive relation with Child–Pugh score, exhibited significantly decreased abundance after splenectomy. At the genus level, 11 genera were differentially abundant between patients and HCs, but 9 genera of them restituted to normal levels by certain degree after splenectomy. PICRUSt analysis showed that the relative abundance of 17 KEGG pathways was partially restored after splenectomy. Four of them were amino acid-related pathways: lysine degradation, tryptophan degradation, amino acid metabolism, and protein digestion and absorption. These findings were supported by metabonomics results which showed that relative abundance of amino acid and corresponding catabolites changed toward normal. In addition to the variations in the relative abundances of bacteria and metabolites, the correlation between them also altered in patients after splenectomy. Dysbiosis in gut microbiome and related metabolism of LC patients was partially corrected after splenectomy. Whether the improved gut microenvironment could prevent LC-related complications and delay the progress of LC is a propitious objective for future study. Trial registration: ChiCTR-OOB-15007409. Registered November 15, 2015.

## Introduction

Liver cirrhosis is an advanced liver disease resulting from acute or chronic liver injury, including alcohol abuse, obesity, and hepatitis virus infection. Hepatitis B virus infection is widespread in China and represents one of the leading causes for LC (Chen et al., 2011). The prognosis for patients with liver dysfunction in decompensated LC is poor. It could lead to number of complications including portal hypertension, variceal bleeding, ascites, bacterial infections, and hepatic encephalopathy (Tsochatzis et al., 2014).

The liver interacts directly with the gut through the hepatic portal and bile secretion systems (Cesaro et al., 2011). Liver dysfunction has many effects on the gut, including impaired motility (Chang et al., 1998), reduced bile flow, and altered secretion of immunoglobulin A and anti-microbial molecules (Lu et al., 2011). All of these effects could lead to dysbiosis in the microbiota (Zeuzem, 2000). Disturbance of gut microbiota is common in LC patients with a feature of increased abundance of potentially pathogenic bacteria concomitant with decreased level of beneficial bacteria (Chen et al., 2011; Qin et al., 2014; Schnabl and Brenner, 2014; Bajaj et al., 2015). Dysbiosis in microbiota can disrupt the metabolic balance in the gastrointestinal tract (Marcobal et al., 2013). Previous studies have reported that LC patients exhibit a distinct metabolic phenotype, such as disordered amino acid metabolism (Huang et al., 2013; Wei et al., 2013, 2016). In turn, the dysbiosis of gut microbiota may contribute to the progress of liver disease (De Minicis et al., 2014; Tilg et al., 2016). The intestinal microflora is the main source of portal LPS and represents an important prerequisite for the development of liver fibrosis in chronic liver injuries (Paik et al., 2003; Isayama et al., 2006; Seki et al., 2007). In addition, the gut microbiota that go through dysbiosis play an important role in the development of LC-related complications, including bacterial infections, the hyperdynamic circulatory state, and hepatic encephalopathy (Garcia-Tsao and Wiest, 2004; Riordan and Williams, 2010).

The spleen is a secondary lymphoid organ that is intricately organized and provides an excellent structural framework for immune responses (Maioli et al., 2007). However, the hypertrophied spleen in LC with portal hypertension could lead to hypersplenism and consequently pancytopenia which act as an excessive portal flow supplier. Surgical splenectomy has been applied to treat splenomegaly and hypersplenism since the 1950s. Splenectomy is often performed in LC patients as a palliative surgical treatment to attenuate thrombocytopenia and leukocytopenia that induced by LC-related hypersplenism (Sugawara et al., 2000; Tomikawa et al., 2002; Kawanaka et al., 2014). The results of recent studies performed in different clinical settings have indicated that splenectomy contributed to improved liver function (Elsebae and Abu-Zekri, 2008; Imura et al., 2010; Ushitora et al., 2011; Nomura et al., 2014; Yamamoto et al., 2015).

The severity of impaired liver function was found to correlate with the degree of microbial dysbiosis. Microbiota in compensated (Child–Pugh A and B) and decompensated (Child–Pugh C) LC patients were significantly different and *Proteobacteria* levels, specifically *Enterobacteriaceae*, were significantly higher in decompensated cirrhotic patients (Qin et al., 2014). Another study showed similar result that Child–Pugh B patients contained a significant reduction of *Bacteroidetes* and increase in *Firmicutes* and *Proteobacteria* compared with the health people. While fecal microbiota component from Child–Pugh A patients had no significant difference from the health people (Wei et al., 2016). These results indicated that normal gut microbiota depend on normal liver function, and improved liver function observed after splenectomy might have helped to restore gut microenvironment.

In this study, with the methods of 16s rRNA gene sequencing and metabolomics analysis, we found substantial differences in gut microbiota and metabolome between LC patients and healthy controls (HCs). Furthermore, 6 months after splenectomy, altered gut microbiota and metabolome of patients partially recovered toward HC. These results obtained indicate that splenectomy treatment partially ameliorated the dysbiosis of gut environment. Whether these benefits could help to prevent or alleviate LC-related complications and delay the progress of LC which represent a target for future study.

## Materials and Methods

### Study Population

There were three study groups, a cross-sectional study was conducted to see if there was any alternation in gut microbiota and metabonomics in LC patients with portal hypertension compared with matched HC. Afterward, a perspective study was performed in the cohort of patients with initial samples before splenectomy (Pre-S) and follow-up samples obtained 6 months after splenectomy (Post-S). LC with portal hypertension was diagnosed by comprehensively reviewing the results of liver biopsies, imaging examinations and laboratory tests in addition to clinical symptoms, physical signs, medical history, progress notes, and associated complications (Procopet and Berzigotti, 2017). Upper gastrointestinal endoscopy was performed in all patients to assess the severity of esophagogastric varices. The Child–Pugh scoring system was used to assess the prognosis of cirrhosis (Pugh et al., 1973). Cases that progressed to hepatic carcinoma, uncontrolled ascites, encephalopathy, or Child–Pugh class C preoperatively and patients suffering from other diseases, such as hypertension and diabetes, were excluded. The control group consisted of healthy volunteer who visited the First Affiliated Hospital of Harbin Medical University for routine physical examination. The liver imaging and liver biochemistry results as well as other physical, blood, urine, and stool test results of all the healthy volunteer were within the normal range. The control subjects and patients had well matched age, sex, and BMI scores. All participants had no history of antibiotic or probiotic use in the previous 3 months before fecal sample collection, they neither had any history of IBD, hypertension, diabetes, obesity, cancer, and gastrointestinal surgery, such as colectomy or gastrectomy.

The clinical indications for splenectomy is: (a) hypersplenism with platelet counts of 50 × 10^9^/L or less (Watanabe et al., 2007; Yamamoto et al., 2015) and have a bleeding tendency due to thrombocytopenia; (b) relieving symptoms caused by an enlarged spleen, possibly including abdominal distension, pain, and fullness or early satiety; and (c) the history of esophageal varices bleeding or risky esophageal varices (Kawanaka et al., 2014). The study protocol was approved by the Ethics Committee of the First Affiliated Hospital of Harbin Medical University. Written informed consent was obtained from all the participants. The study conformed to the ethical guidelines of the 1975 Declaration of Helsinki and was registered in the Chinese Clinical Trial Registry on November 15, 2015 (ChiCTR-OOB-15007409).

### Sampling, DNA Extraction, and PCR Amplification

Each participant provided a fresh stool sample in hospital that was delivered immediately to the laboratory with insulated box. Upon collection, the fecal sample was immediately divided into aliquots that were then frozen on dry ice and stored at -80°C until the next step. Microbial DNA was extracted from feces using an E.Z.N.A.^®^ Stool DNA Kits (Omega Bio-Tek Inc., Norcross, GA, United States) according to the manufacturer’s instructions. The V3–V4 hypervariable regions of the bacterial 16s rRNA gene were amplified using the following primer pairs: forward 341-CCTAYGGGRBGCASCAG and reverse 806-GGACTACNNGGGTATCTAAT in a thermocycler PCR system (GeneAmp 9700, ABI, United States) (for details refer to the Supplementary Material).

### 16s rRNA Gene Sequencing

Purified amplicons were pooled in equimolar and sequenced on an Illumina MiSeq platform (Illumina, San Diego, CA, United States) with PE300 mode according to the standard protocols provided by Majorbio Bio-Pharm Technology Co. Ltd. (Shanghai, China) (detailed methods are provided in the Supplementary Material).

### UPLC–MS

Metabolism analysis was performed using a Waters ultra performance liquid chromatography (UPLC) system equipped with a binary solvent delivery manager and a sample manager coupled with a Waters Q-TOF Mass spectrometer (MS) equipped with an electrospray ionization (ESI) source which is capable of operating in both positive or negative ion modes (Waters Corporation, Milford, MA, United States). Standardized samples for quality control were prepared by mixing aliquots of all samples into a pooled sample and then analyzed using the same method. The five QCs were injected at every six samples throughout the analytical run to obtain a set of data from which repeatability could be assessed (detailed methods are provided in the Supplementary Material).

### Bioinformatic and Statistical Analyses

16s rRNA gene sequencing data were processed using the Quantitative Insights Into Microbial Ecology platform (QIIME; V.1.9.1) (Kuczynski et al., 2012). OTUs were picked using a cut-off of 97% similarity, and the identified taxonomy was then aligned using the Greengenes database (V.13.8). Chimeric sequences were identified and deleted. A rarefaction curve was constructed using the OTUs and Sobs index to prevent methodological artifacts originated from variations in sequencing depth. α-Diversity was measured according to species richness based on the observed OTUs number with the indices of sobs and Chao1, and the results were displayed Origin 2017 software (OriginLab Corp., Northampton, MA, United States) to compare bacterial richness and diversity across samples. β-Diversity was estimated by computing the Weighted UniFrac distance and visualized using principal coordinate analysis (PCoA), and the results were plotted using the WGCNA, stats, and ggplot2 package in R software (Version 3.4.4). Only bacterial taxa with average abundances >1% were compared between groups in the significant difference analysis using Wilcoxon rank-sum test.

Functional composition profiles of the gut metagenomes were predicted from 16s rRNA gene sequences using PICRUSt in the form of level III KEGG database pathways (Langille et al., 2013). Pathways that are present in >10% of the samples were brought into comparison analysis using Wilcoxon rank-sum test. Spearman’s rank correlation was used to evaluate the correlation coefficient between KEGG pathway and bacteria taxa.

UPLC/MS raw data were analyzed using progenesis QI software (Waters Corporation, Milford, MA, United States). The positive and negative data were combined and imported into the SIMCA-P+ 14.0 software package (Umetrics, Umea, Sweden). A principle component analysis (PCA) was performed to visualize metabolic alterations among groups. Metabolites were identified by comparing observed accurate mass and MS/MS spectra to accurate mass data and spectra available in online databases including METLIN database, human metabolite database, and progenesis QI (Waters Corporation, Milford, MA, United States). Metabolites compared between groups in the significant difference analysis using Wilcoxon rank-sum test. A heatmap of the key metabolites identified in this process was constructed using pheatmap package in R.

### Correlation Network Analysis

Correlation networks were generated using data for bacterial genera, metabolites, and serum indexes for liver impairment (ALT, AST, PT, PT.A, TBIL, and TBA) which were identified to be significantly correlated by Spearman’s rank correlation coefficient (Student’s *t*-test, *p* < 0.05, correlation coefficient >0.6). For the prospective study, a correlation network was calculated to extract edge with only correlation differences between Pre-S and Post-S groups (*p* < 0.05, Student’s *t*-test) and where at least one of the original Spearman correlation was >0.6 (Morgenthal et al., 2006). The correlations and corresponding attributes within the network model were visualized by Cytoscape (Shannon et al., 2003). We then compared the network topology to identify sub-networks on specific bacteria which may be significantly related with recovery of normal liver functions (Assenov et al., 2008).

All statistical analyses were performed using R package and SPSS 19.0 software. An independent-sample hypothesis test was used between Pre-S and HC groups in the cross-sectional study, while the paired-sample hypothesis test was used between Post-s and Pre-s groups in the perspective study. Multiple hypothesis tests were adjusted using the Benjamini and Hochberg FDR, significant differences were considered when results were below an FDR threshold of 0.05. All tests for significance were two-sided and *p*-values < 0.05 were considered significant.

## Results

### Study Population

Recruitment and sampling of the participants were carried out at the First Affiliated Hospital of Harbin Medical University. From December 1, 2015 to December 31, 2016, 44 patients who need therapeutic splenectomy were assessed for enrolment. Twenty-five patients were excluded based on the inclusion–exclusion criteria preoperatively. At the time point of 6 months after splenectomy, one patient had progressed to hepatocellular carcinoma and six patients had taken antibiotics within 3 months before fecal sample collection was excluded. Finally, fecal samples were analyzed for 16 HCs (the HC group) and 12 patients both before (Pre-S group) and after (Post-S group) splenectomy. Etiology underlying the LC was hepatitis B virus infections (*n* = 7), hepatitis C virus infections (*n* = 1), alcoholic LC (*n* = 2), and autoimmune LC (*n* = 2), respectively. Six months after splenectomy, all patients had reverted back to their normal diet and living habits, BMI index of patients was not changed significantly compared with preoperative data (Wilcoxon rank-sum test, *p* = 0.88, **Table 1**). Demographic and clinical characteristics of the participants were summarized in **Table 1**. As expected, LC patients exhibited marked impairments in liver function and blood counts compared to HC subjects, and such deleterious alterations were ameliorated after splenectomy. Child–Pugh scores decreased from 6.2 ± 0.9 to 5.1 ± 0.3 (Wilcoxon rank-sum test, *p* < 0.01), with 4 out of 12 patients who were preoperatively classified as Child–Pugh class B being postoperatively improved to class A. The data obtained from the participating patients during perioperative period were presented in Supplementary Table S1. We performed laparoscopic splenectomy in five patients and open splenectomy in seven.

**Table 1:** Information of pre- and post-splenectomy patients compared with health control.

	LC		
				
	Pre	Post	Control		
			
	*n* = 12	*n* = 12	*p*-value	*n* = 16	*p*-value
Age	45.4 ± 9.7			47.0 ± 8.5	0.72
Male/female	7/5			9/7	>0.99
BMI (kg/m^2^)	23.2 ± 0.8	23.3 ± 0.9	0.88	23.1 ± 1.0	0.75
Total bilirubin (μmol/L)	31.1 ± 27.5	17.9 ± 8.3	0.09	11.1 ± 4.3	
Albumin (g/L)	39.4 ± 5.7	42.7 ± 3.5	0.02	42.3 ± 3.4	
Prothrombin time (s)	13.8 ± 1.1	12.0 ± 0.6	<0.01	12.2 ± 0.5	
Prothrombin activity	57.4 ± 15.0	79.0 ± 8.6	<0.01	76.0 ± 8.8	
ALT (U/L)	52.7 ± 75.0	36.6 ± 14.2	0.49	15.3 ± 10.3	
AST (U/L)	77.7 ± 126.1	47.1 ± 17.5	0.43	19.3 ± 6.2	
Total bile acids (μmol/L)	42.1 ± 25.7	20.2 ± 10.4	<0.01	5.1 ± 3.1	
White blood cell count (10^9^/L)	2.0 ± 0.5	5.2 ± 1.0	<0.01	6.5 ± 2.6	
Platelet count (10^9^/L)	44.2 ± 17.6	340.5 ± 154.4	<0.01	266.1 ± 43.2	
Ascites	5(42)	1(8)			
Encephalopathy	0(0)	0(0)			
Child–Pugh score (points)	6.2 ± 0.9	5.1 ± 0.3	<0.01		
Child–Pugh class (A/B/C)	8/4/0	12/0/0			


*Student’s *t*-test was used to compare age and BMI between LC (*n* = 12) and control (*n* = 16). Fisher’s exact test was used to compare gender distribution between LC and control. Paired Student’s *t*-test was used to compare clinical indices of LC before and after splenectomy. Values are expressed as the means ± SD. Numbers in parenthesis are %. ALT, alanine transaminase, AST, aspartate aminotransferase, BMI, body mass index.*

### Gut Microbiota in Patients Was More Similar With Health Control After Splenectomy

**FIGURE 1:**
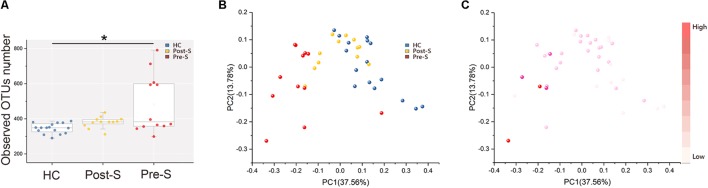
Microbiota diversities in Pre-S and Post-S compared with HC. **(A)** α-Diversity illustrated by microbiota richness [number of observed taxonomic unit (OTU) in patient Pre-S (*n* = 12), Post-S (*n* = 12) and HC (*n* = 16)]. Boxes represented the 25–75th percentile of the distribution; the median was shown as a thick line in the middle of the box; whiskers extend to values with 1.5 times the difference between the 25th and 75th percentiles Wilcoxon rank-sum test, ^∗^*p* < 0.05. **(B)** Principal coordinate analysis (PCoA) of Weighted UniFrac distance from three groups. **(C)** The same PCoA plot as **(B)**, colored by α-diversity measured by the number of observed OTU.

After filtering, an average of 68,802 reads per sample was obtained (58,471–80,984). Sample size was equalized to 58,471 for each sample using random subtraction (Supplementary Table S3). First, sequencing depths were examined by plotting the rarefaction curve for richness (sobs index) and the numbers of shared OTUs (Supplementary Figure S1). Most of the samples reached plateaus, suggesting that sequencing depth was adequate. α-Diversity was assessed according to observed OTU numbers, and the data revealed that gut microbiota richness was higher in the Pre-S group than in the HC group (Wilcoxon rank-sum test, *p* < 0.05; **Figure 1A**), The OTU number was lower in Post-S group compared with Pre-S group but the difference was not statistically significant yet (Wilcoxon rank-sum test, *p* = 0.15; **Figure 1A**). However, the result of Shannon diversity index that measures both richness and evenness was the highest in the Post-S group (Supplementary Figure S2). PCoA based on Weighted UniFrac distances revealed that the bacterial composition of Pre-S group was clearly segregated from that of HC group. After splenectomy, the bacterial composition of Post-S group experienced obvious changes and become more similar with HC group (**Figure 1B**). Such finding was supported by the comparison of taxonomic distribution of all three groups at levels of phylum, family, and genus (Supplementary Figure S3). Notably, we found that OTU number can explain the differences along the first principal coordinate (**Figure 1C**).

**FIGURE 2:**
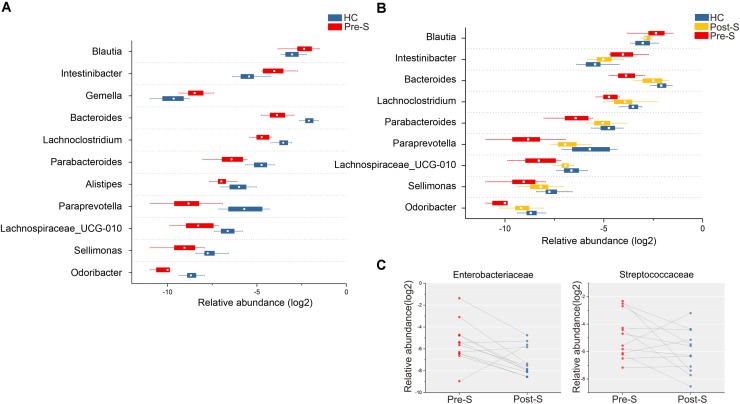
Microbiome phylotype alterations. **(A)** The relative abundance of 11 genera was significantly different between patients (*n* = 12) and HC (*n* = 16), Wilcoxon rank-sum test with FDR correction, *p* < 0.05. **(B)** The microbiome composition was partially mitigated in Post-S (*n* = 12), nine of the patient-associated genera were reversed after splenectomy in patients, compared with Pre-S group, paired Wilcoxon rank-sum test, *p* < 0.05. The relative abundance of the genus was plotted on a logarithmic scale. Box plot illustration was provided in **Figure 1**. **(C)** Comparison of the relative abundance of *Enterobacteriaceae* and *Streptococcaceae* from “Pre-S” (*n* = 12) and “Post-S” (*n* = 12) groups, connected by lines, pairs of Pre-S and Post-S. Significant higher abundance was found in “Pre-S” group, paired Wilcoxon rank-sum test, *p* < 0.05.

Bacterial phylotypes those were significantly different between groups were identified. At the phylum level, there were significantly fewer *Bacteroidetes* in the Pre-S group compared to the HC, while *Firmicutes* and *Proteobacteria* were over-represented in Pre-S group (Wilcoxon rank-sum test, *p*_fdr_ < 0.05). The relative abundance of *Bacteroidetes* was significantly increased, while that of *Proteobacteria* was significantly decreased in Post-S group (Wilcoxon rank-sum test, *p* < 0.05; Supplementary Figure S4). At the family level, *Enterobacteriaceae* and *Streptococcaceae*, two kinds of taxa comprising many pathogenic species, were enriched in patients (Wilcoxon rank-sum test, *p*_fdr_ < 0.05, Supplementary Figure S5) and positively correlated to Child–Pugh scores (Spearman’s rank correlation, *r* = 0.38, *p* < 0.05, *r* = 0.46, *p* < 0.01, respectively; Supplementary Figure S6); however, the two taxa were significantly suppressed after splenectomy (Wilcoxon rank-sum test, *p* < 0.05; **Figure 2C**).

At the genus level, 11 genera were differentially abundant between Pre-S group and HC group (Wilcoxon rank-sum test, *p*_fdr_ < 0.05; **Figure 2A**). Among the nine HC enriched genera, *Bacteroides*, *Lachnoclostridium*, *Parabacteroides*, *Paraprevotella*, *Lachnospiraceae_UCG-010*, *Sellimonas*, and *Odoribacter* displayed significantly increased abundance after splenectomy (Wilcoxon rank-sum test, *p* < 0.05). Of the two genera that were enriched in Pre-S group, *Intestinibacter* and *Blautia* were significantly decreased after splenectomy (Wilcoxon rank-sum test, *p* < 0.05; **Figure 2B**). HC enriched *Blautia*, *Lachnoclostridium*, *Lachnospiraceae_UCG-010*, and LC enriched *Sellimonas*, four genera were all affiliated to *Lachnospiraceae* family, this may explain why we didn’t see any difference in abundance of *Lachnospiraceae* family between HC and Pre-S group (Wilcoxon rank-sum test, *p*_fdr_ > 0.05, Supplementary Figure S5).

### Microbial Functions Were Partly Recovered in Patients After Splenectomy

**FIGURE 3:**
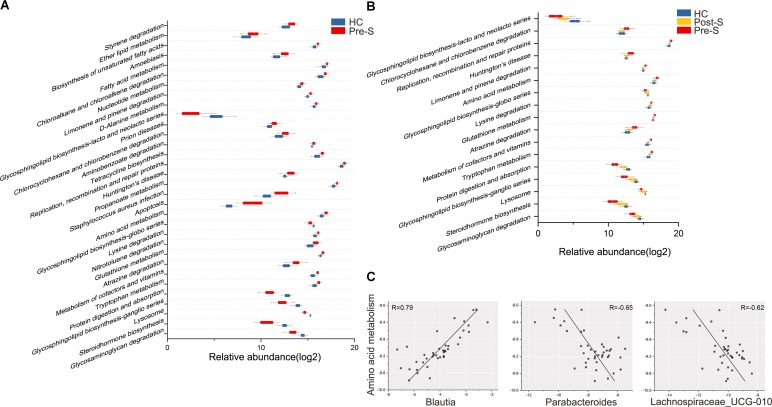
Alternation of predicted microbial functional composition from 16s rRNA sequencing data with PICRUSt. **(A)** Significantly different abundance of 32 KEGG pathways (level III) between patients (*n* = 12) and HC (*n* = 16). Wilcoxon rank-sum test with FDR correction, *p* < 0.05. **(B)** Comparison of the KEGG pathways between “Pre-S” group (*n* = 12) and “Post-S” group (*n* = 12). Same as **Figure 2**, 17 pathways reversed significantly in patients after splenectomy. Paired Wilcoxon rank-sum test, *p* < 0.05. **(C)** Relative abundance of amino acid metabolism pathway was positively correlated with the LC-enriched *Blautia* (*r* = 0.79); negatively correlated with the healthy enriched *Parabacteroides* (*r* = –0.65) and *Lachnospiraceae_UCG-010* (*r* = –0.62), Spearman’s rank test, *p* < 0.05.

Biofunctions of the gut microbiota were predicted from 16s rRNA gene sequencing data using PICRUSt. A multiple analysis of KEGG pathways categories (level III) with PCA showed that Post-S group could be separated from Pre-S group but resembled HC group (Supplementary Figure S7). Thirty-two pathway categories were considerably different between Pre-S group and HC group (Wilcoxon rank-sum test, *p*_fdr_ < 0.05; **Figure 3A**) and 17 of them were partially restored after splenectomy (Wilcoxon rank-sum test, *p* < 0.05; **Figure 3B**).

Four of the 17 pathways were related to amino acid, “lysine degradation,” “tryptophan degradation,” and “amino acid metabolism” pathways were higher in Pre-S group than HC group, while the pathway of “protein digestion and absorption” was in the opposite, all these differences were minimized after splenectomy. Further analysis revealed that the abundance of “amino acid metabolism” pathway positively correlated with LC-enriched genus *Blautia* (Spearman’s rank correlation, *r* = 0.79, *p* < 0.05), but negatively correlated with the healthy-enriched genera *Parabacteroides* (Spearman’s rank correlation, *r* = -0.65, *p* < 0.05) and *Lachnospiraceae_UCG-010* (Spearman’s rank correlation, *r* = -0.62, *p* < 0.05; **Figure 3C**).

### Gut Metabolism Was Improved in Patients After Splenectomy

**FIGURE 4:**
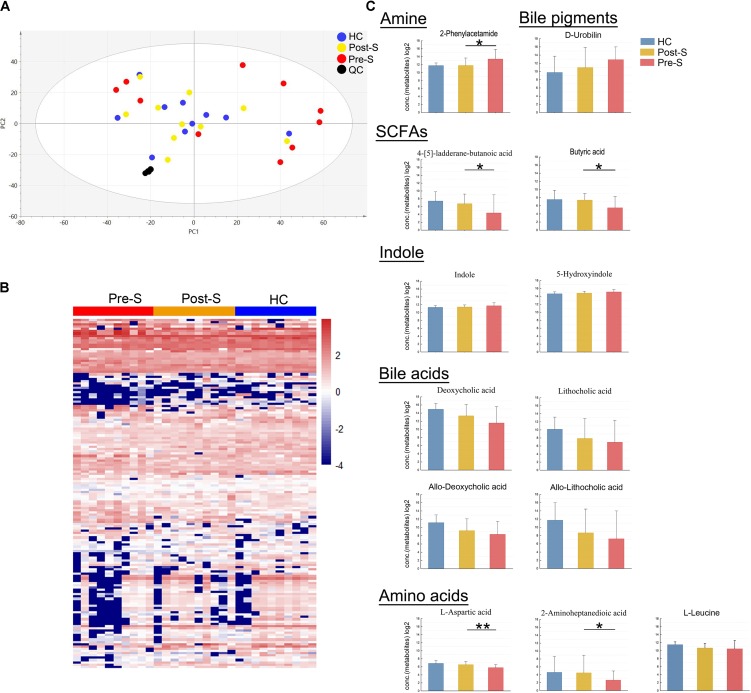
Characteristics of metabolites. **(A)** PCA of all metabolites concentrations of “Pre-S” group (*n* = 10), “Post-S” group (*n* = 10), and “HC” group (*n* = 10). **(B)** Color-coded heat map displaying the related metabolites amounts in three groups. The color scale represented the scaled abundance of each metabolite, denoted as *Z*-score, with red and blue indicated increased and decreased concentrations, respectively. **(C)** Related common sets of metabolic perturbations. LC-related metabolites that significantly different between health control and pre-splenectomy patient. Wilcoxon rank-sum test with FDR correction, *p* < 0.05. The metabolic was partially mitigated in post-splenectomy patients. The values displayed are the average concentrations of log2 of each metabolite. Data are shown as mean values ± SD for each group. ^∗^*p* < 0.05, ^∗∗^*p* < 0.01.

Typical base peak intensity chromatograms (BPC) of fecal extracts were different among the three groups (*n* = 10 each groups, Supplementary Figure S8). A total of 4401 detectable peaks were observed in UPLC/MS positive and negative modes after the peaks representing internal standards and any known pseudo-positive peaks were removed. Similarly, less difference between the HC and Post-S groups was found compared with that between the HC and Pre-S groups showed by PCA analysis (**Figure 4A**). To further explore the impact of splenectomy on fecal metabolism, using the identified significant metabolites, we also drew the heatmap plot to classify scaled abundance of 4406 metabolites. As illustrated in **Figure 4B**, the samples from Post-S group were clearly distinct from those in Pre-S group but were similar to those in the HC group. A total of 391 peaks were found to be significantly different between Pre-S groups and HC groups (Wilcoxon rank-sum test, *p*_fdr_ < 0.05), and a subset (157) of metabolites was successfully identified (Supplementary Table S4).

Next, we assessed the relative abundance of 13 kinds of important metabolites from them and found that specific changes had occurred after splenectomy (**Figure 4C**). These 13 metabolites belonged to bile acid, SCFAs, amino acid, catabolites of amino acid, and urobilin, all these metabolites had changed toward normal level after splenectomy in the Post-S group and five of them achieved statistical significance compared with the Pre-S group (Wilcoxon rank-sum test, *p* < 0.05, **Figure 4C**). Three identified amino acids were originally lower in Pre-S groups but two of them, L-aspartic acid and 2-aminoheptanedioic acid, had significantly increased in the Post-S group compared with Pre-S group (Wilcoxon rank-sum test, *p* < 0.01 and *p* < 0.05, respectively). The content of one catabolite of phenylalanine, 2-phenylaceta (amine), was increased in Pre-S group but decreased significantly in Post-S group (Wilcoxon rank-sum test, *p* < 0.05), while another two amino acid catabolites, indole and 5-hydroxyindole, also were higher in Pre-S group but decreased in Post-S group although without significance (Wilcoxon rank-sum test, *p* > 0.05). These results were consistent with KEGG pathway analysis of amino acid metabolism as described above, which showed that the abundance of the amino acid metabolism-related pathways was originally higher in Pre-S group but decreased after splenectomy. Another group of metabolites were bile acids that were lower in Pre-S groups (i.e., deoxycholic acid, allo-deoxycholic acid, lithocholic acid, and allo-lithocholic acid). After splenectomy, all of them increased although without statistical significance (Wilcoxon rank-sum test, *p* > 0.05). D-Urobilin, a catabolite of bile pigment that is normally present at a high concentration in Pre-S group, was found decreased in Post-S group (Wilcoxon rank-sum test, *p* = 0.769). It might have resulted due to serum TBIL level which was obviously higher in Pre-S group but decreased in Post-S group although without significance (Wilcoxon rank-sum test, *p* = 0.09, **Table 1**). We had identified two SCFAs compounds, 4-[5]-ladderane-butanoic acid and butyric acid which were presented at low concentrations in Pre-S group, but significantly increased in Post-S group (Wilcoxon rank-sum test, *p* < 0.05, **Figure 4C**).

### Correlation Network Analysis

The findings that structure and metabolism of gut microbiota in LC patients changed after splenectomy and recovered toward HCs prompted us to investigate how the changes of microbial components correlated with and affected gut metabolism and how splenectomy influenced such correlations. Correlation were analyzed between 4401 metabolites, 485 genera, and 5 serum parameters.

The global correlation networks consisted of 80,681 correlations (2248 features) in HC group, 92,615 correlations (2242 features) in Pre-S group, and 85,296 correlations (2245 features) in Post-S group (Supplementary Figure S9). The average number of neighbors was 71.955 in HC group, 82.742 in Pre-S group, and after splenectomy it was decreased to 76.433 in Post-S group (Supplementary Table S2). These parameters showed that Pre-S group had more complex metabolic network compared to HC group, the metabolic network became less complex in Post-S group after splenectomy and closed to HC, indicating that splenectomy has a major effect on the metabolic network, reducing a number of the metabolic interactions, while keeping the nodes themselves intact. Next, we constructed a correlation difference network which provided a global view of correlations that was significantly altered between Pre-S and Post-S group (Student’s *t*-test, *p* < 0.05, Supplementary Figure S10). This network contains only 1890 features and 4140 correlations, which is substantially smaller than the original network with only 83% of the original features. Number of correlations can be conveniently seen involving bacteria (yellow squares) and serum indexes (blue squares).

**FIGURE 5:**
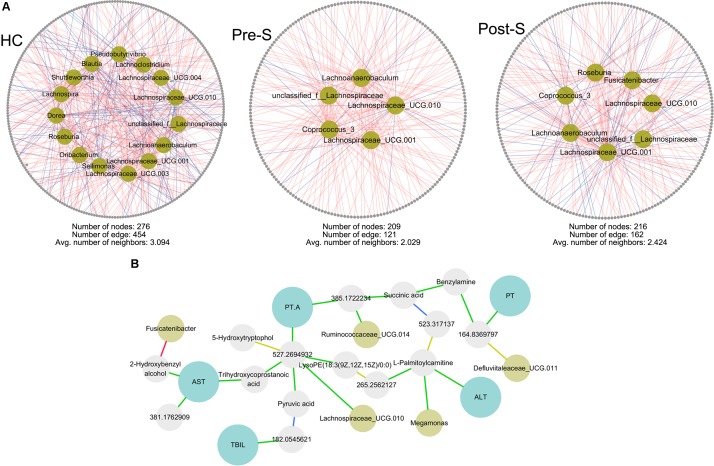
Correlation network analysis between bacteria (yellow nodes), metabolites (gray nodes), and clinical parameters (blue nodes). **(A)** Network center on genera affiliated to *Lachnospiraceae* family. The complex correlation network represented parameters that were linked negative (blue edge) or positive (red edge) with a correlation coefficient >0.6, *p* < 0.05. **(B)** Subnetwork from correlation difference network that center on clinical parameters. Blue edges demonstrate linkages that were positive in the Pre-S group but became negative in Post-S; red edges represent correlations that changed from negative to positive; green and yellow edges represent correlations that keep positive and negative, respectively, but with significant change (Student’s *t*-test, *p* < 0.05). ALT, alanine transaminase; AST, aspartate aminotransferase; PT, prothrombin time; PT.A, prothrombin activity; TBIL, total bilirubin.

*Lachnospiraceae* family were not found to have significant difference between HC and Pre-S group, but the genus affiliated to this family were differently distributed. So we were curious to know the functional change of *Lachnospiraceae* in LC patient before and after splenectomy. Correlation network was constructed from three groups that covered 4401 metabolites and 50 genera which affiliated to *Lachnospiraceae*. Different to the global network, HC group possessed the most complicated network that harbors 15 genera, 454 correlations between 256 features. By contrast, network of Pre-S group only had 5 genera, 208 correlations between 121 features. The network feature of Post-S group was in middle place, 7 genera and 216 correlations between 162 features (**Figure 5A**). These results manifested that bacterial metabolic function of *Lachnospiraceae* was obviously different in Pre-S group compared with HC group but changed toward HC in Post-S after splenectomy rather than overall population in *Lachnospiraceae* family.

Subnetwork center on serum parameters from correlation difference network showed that all the corrections that link serum indexes were keep positive but with significant coefficient change before and after splenectomy, although there is no direct correlation between serum indexes and bacterial genus (**Figure 5B**).

## Discussion

In the current study, we applied 16s rRNA gene sequencing and UPLC/MS to evaluate gut microbiota and metabolome. The cross-sectional study showed that LC was associated with altered gut microbiota composition, function, and metabolism, which were in line with the results from several previous studies (Chen et al., 2011; Ushitora et al., 2011; Marcobal et al., 2013; Qin et al., 2014; Schnabl and Brenner, 2014; Bajaj et al., 2015). In our prospective study, gut microbial dysbiosis, metabolic disorder, and impaired liver function were found partially mitigated half year after splenectomy.

We found that substantial differences in the gut microbial composition between patients and HCs were very evident. The difference became less obvious after splenectomy. At the phylum level, *Bacteroidetes* were significantly under-represented, whereas *Firmicutes* and *Proteobacteria* were over-represented in patients, in accordance with the results of a previous study which made comparison between LC patients and healthy people by 16s rRNA gene sequencing (Chen et al., 2011). After splenectomy, *Bacteroidetes* increased while *Proteobacteria* decreased significantly. *Enterobacteriaceae* and *Streptococcaceae*, the two LC-enriched families which contain many pathogenic species and positively correlated to Child–Pugh score were significantly suppressed after splenectomy significantly.

The mechanisms underlying the alleviation of liver impairment after splenectomy remain controversial (Ueda et al., 2003; Lesurtel et al., 2006; Yada et al., 2015). TGF-β, released from enlarged spleen, has been shown to exert inhibition of liver regeneration. Moreover, TGF-β exaggerates liver fibrosis and apoptosis (Ueda et al., 2003). Hence, inhibition splenectomy facilitated inhibition of TGF-β production which led to alleviation of chemically induced LC (Akahoshi et al., 2002). Splenectomy up-regulates TNF-α then reduces dimethylnitrosamine (DMN)-induced liver damage (Murata et al., 2001). Recent studies have shown that recovery of thrombocytopenia may positively be followed by liver function improvement for the reason that platelet-derived serotonin is important in liver regeneration (Lesurtel et al., 2006). The hepatic accumulation of Ly-6Clo macrophages/monocytes might have played significant roles in the tissue remodeling process in cirrhotic livers after splenectomy (Yada et al., 2015).

Studies that have been done before pointed that the severity of liver function impairment was correlated with the degree of microbial dysbiosis. Liver dysfunction can cause reduced bile flow and reduced bile acids in gut (Lu et al., 2011). Bile acids are important not only for facilitating dietary fats and vitamins absorption, but are also ligands for the nuclear receptor PAMPs (FXR) and the G-protein-coupled receptor TGR5. BAs do have direct bacteriostatic effects, and so intestinal bacterial overgrowth could also be a result of decreased total fecal bile acids in LC patients (Kakiyama et al., 2013). Conjugated bile acids bind to FXR in intestinal epithelial cells, which increases production of the antimicrobial proteins and prevents bacterial overgrowth and promotes epithelial cell integrity (Inagaki et al., 2006). The FXR agonist obeticholic acid has been shown to reduce markers of liver inflammation and fibrosis in patients (Mudaliar et al., 2013). Together with TBA, the ratio between secondary and primary fecal bile acids is also decreased in LC (Kakiyama et al., 2013). In this study, LC patients showed reduction in families belonging to the order *Clostridiales*, including *Lachnospiraceae* and *Ruminococcaceae* although without statistical significance after FDR adjust. These groups are capable of performing 7α-dihydroxylation (Kakiyama et al., 2013), metabolizing primary bile acids into secondary bile (Ridlon et al., 2006). The relative abundance of *Ruminococcaceae* increased significantly after splenectomy. We had identified four kind of bile acids, deoxycholic acid, lithocholic acid, allo-lithocholic acid, and allo-deoxycholic acid, all of them were secondary bile acids and found increased after splenectomy. These obtained results supported the notion that the outcome of improved liver function after splenectomy might have contributed to gut microbiota improvement.

Gut directly interacts with liver via the hepatic portal and bile secretion systems (Cesaro et al., 2011). Microbiota and metabolomics improvement in gut may contribute to reduced liver impairment and LC-related complication in turn.

The intestinal microflora is the main source of portal LPS and represents an important prerequisite for the development of liver fibrosis in chronic liver injuries (Paik et al., 2003; Isayama et al., 2006; Seki et al., 2007). Several mechanisms contribute to the intestinal translocation of PAMPs in patients with cirrhosis. These include a leaky intestinal barrier and immune deficiency, and some of these mechanisms are known to be closely related to the composition of the intestinal microbiome (Winer and Ploss, 2016). Interestingly, bacteria from *Enterobacteriaceae* family (including *Escherichia coli*, *Klebsiella*, *Proteus*, and *Enterobacter*) are all regarded as PAMPs-producing bacteria and were increased in the microbiota of LC patient in both ours and previously conducted studies and found to be decreased after splenectomy in our study (Chen et al., 2011; Lu et al., 2011; Bajaj et al., 2012a,b). It is also well recognized that dysbiosised gut flora plays an important role in LC-related complications, including bacterial infections, the hyperdynamic circulatory state, and hepatic encephalopathy (Garcia-Tsao and Wiest, 2004; Riordan and Williams, 2010; De Minicis et al., 2014; Tilg et al., 2016). The enrichment of these potentially pathogenic phylotypes is in accordance with previous research obtained from bacteriological culture of fecal samples, in which patients who had cirrhosis with minimal hepatic encephalopathy had significant fecal overgrowth of *E. coli* and *Staphylococcus* sp. (Liu et al., 2004). As concluded by many clinical studies, *E. coli* and *Streptococcus* are the main causes of bacterial infection in patients with cirrhosis (Riordan and Williams, 2006). Probiotics, prebiotics, and antibiotics aimed at manipulating gut microflora have shown the ability to reduce rates of bacterial infection and hepatic encephalopathy in LC patients (Novella et al., 1997; Grange et al., 1998; Liu et al., 2004). These results indicated that the mitigated gut microbiota dysbiosis after splenectomy may help alleviated liver impairment and LC-related complications.

PICRUSt analysis showed that the enrichment in pathway of “lysine degradation,” “tryptophan degradation,” and “amino acid metabolism” in patient was partially reversed after splenectomy. These results were supported by metabolome study showing that fecal samples of the patients contained higher abundance of amines and indoles but lower abundance of amino acids than those in HCs, similar to the findings of a previous study that suggested cirrhotic patients were enriched in amino acid transport and metabolic products (Wei et al., 2013, 2016). However, our study showed that the metabolic disturbance of amino acids had improved after splenectomy in affected patient. L-Leucine, which was increased after splenectomy in the patients, is a branched-chain essential amino acid, BCAA. A series of studies indicate that in advanced cirrhosis, oral supplementation with BCAA may improve cognitive function especially in patients with chronic HE (Marchesini et al., 1990). In addition, BCAA may have specific effects on liver function; two large randomized trials have generated evidence that oral BCAA slowed the progression of advanced cirrhosis and prolonged event-free survival (Marchesini et al., 2003; Muto et al., 2005). Intestinal bacteria metabolize tryptophan into indole which was found decreased after splenectomy in LC patient, is then further metabolized into oxindole, a sedative compound putatively involved in the pathophysiology of hepatic encephalopathy (Riggio et al., 2010). These results support that the recovery of “amino acid metabolism” pathway brings about decreased indole, increased L-lysine in the gut of LC patients after splenectomy which might contribute largely to prevent hepatic encephalopathy and slow the progress of liver impairment.

Moreover, *Lachnospiraceae_UCG.010*, *Lachnobacterium*, and *Sellimonas* which belong to the family *Lachnospiraceae* was found decreased in LC patients but increased significantly after splenectomy. These group of genera were known to benefit from participating in carbohydrate fermentation into SCFAs and gases (CO_2_ and H_2_) in the human intestine (Duncan et al., 2007). The suppression of these fermentation-related bacteria could cause a decline in SCFAs production. These results were consistent with a metabolomics study which showed that low abundance of 4-[5]-ladderane-butanoic acid and butyric acid in LC patients was increased after splenectomy. SCFAs are significant energy sources for gut enterocytes, influencing the gastrointestinal barrier function to reduce pathogenic bacterial colonization (Brahe et al., 2013). A decrease in SCFAs would result in increased colonic PH, increased ammonia production, and absorption in the gut (Vince et al., 1990), with hyperammonemia being a very important pathogenetical factor in hepatic encephalopathy.

The recovery observed in the gut microenvironment following splenectomy reflects changes in not only the abundances of bacteria and metabolites but also in the correlations between them. The correlation network characters changed toward HC in patients after splenectomy. We hypothesized that correlations that were present in one group but missing or changed in another may offer potential clues to the functionality of the system. Our data supported the notion that bacterial metabolic functions were altered in addition to bacterial abundance.

Earlier study has found that *Lachnospiraceae* was decreased in LC patients and correlated negatively with Child–Pugh score (Imura et al., 2010; Bajaj et al., 2012b). In this study, there was no significant difference in the abundance of *Lachnospiraceae* family between Pre-S and HC group. Subnetwork focus on genera that belonged to *Lachnospiraceae* family showed that HC group possessed more complicated network compared with Pre-S group and the gap became small after splenectomy in Post-S group. These results manifested that bacterial metabolic function can change rather than overall population in *Lachnospiraceae* family. The serum indexes for liver impairment: AST, ALT, TBIL, PT, and PT.A were found keep positive correlated with gut metabolites but the correlation coefficient was significantly changed in patients after splenectomy. These correlation differences are key in discovering the relationship between liver function improvement and gut microenvironment variation in patients before and after splenectomy.

After splenectomy, a recovery of the composition and metabolism of the microbiota was observed in this study. First, before–after study results could have been influenced by effects such as environmental and unpredictable factors which is a common issue in this kind of study. In this study the BMI and diet, two main factors which had been showed to relate microbiota (Cotillard et al., 2013), were kept consistent before and after splenectomy. Second, a previous study was done on LC and gut microbiota, which enrolled patients with various etiologies. The study showed that microbiota difference existed between patients with different etiologies (Chen et al., 2011; Bajaj et al., 2014; Qin et al., 2014). Due to the limited sample size, sub-classification analysis based on different etiology cannot be conducted in this study, so we cannot exclude that the underlying etiology is a relevant cofounding factor in this study. Further taking the study into account, the etiology needs to be conducted to address this issue. Last, our pathway analysis was based on the inferred meta-genome of the 16s rRNA gene sequence, and while inferring a metagenome (i.e., in PICRUSt) is a common approach in studies that use 16s rRNA gene, but short-gun sequencing of metagenomics and metatranscriptomics could potentially reveal more accurate information related to the composition of the microbial community and its functions.

**FIGURE 6:**
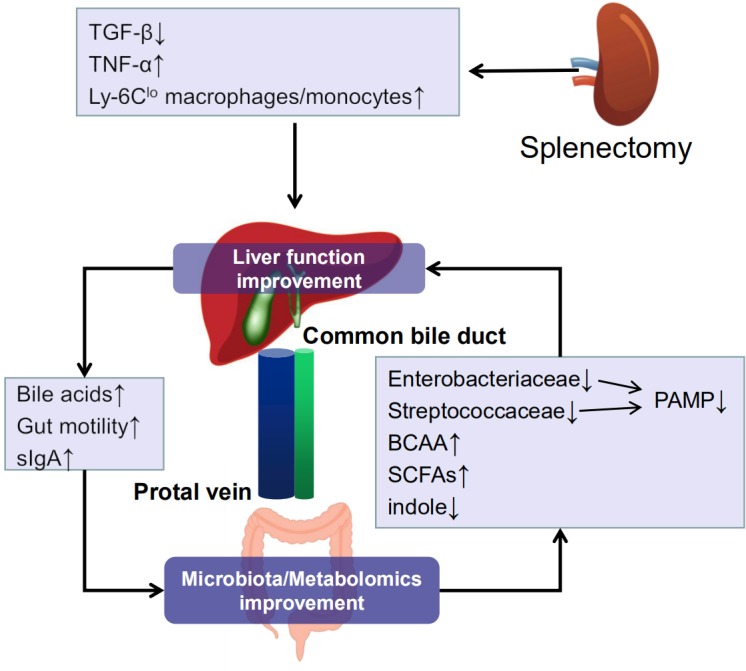
Interaction between liver and gut. Splenectomy led to liver function improvement, which could remit gut microbiota and metabolomics dysbiosis. In turn, better gut microenvironment could alleviate liver impairment.

However, given all these limitations, we were still able to show that LC patients who received splenectomy exhibited benefits that include improved liver function in addition to the recovery of the gut microenvironment. The mechanisms underlying these effects are not clear at this stage; as summarized in **Figure 6**, we suggested that the liver function and the gut microenvironment are likely to affect each other, and the normal microenvironment of gut depended on normal liver function. Splenectomy results in decreased relative abundance of pathogenic bacteria such as *Enterobacteriaceae* and *Streptococcaceae* as well as catabolite of amino acid, such as indole. Also, an increase of BCAA namely, L-leucine in LC patients. Whether and how these changes, after splenectomy, help avoid LC-related complications and delay the progress of LC represent a target for future study.

## Data Sharing Statement

Raw data for 16s-rRNA amplicon sequencing and UPLS/MS are available upon request.

## Author Contributions

YW and YL made substantial contributions from conception of the experiment to design and acquisition of data. JL and YJ collected the samples. YW, AL, JL, and LZ carried out the clinical diagnosis and treatment. YL, FZ, and JF performed the bioinformatics, statistical analysis, and interpreted data. All authors read and approved the final manuscript.

## Conflict of Interest Statement

The authors declare that the research was conducted in the absence of any commercial or financial relationships that could be construed as a potential conflict of interest.

## Abbreviations

ALT: alanine transaminase
AST: aspartate aminotransferase
BMI: body mass index
FDR: false discovery rate
KEGG: Kyoto Encyclopedia of Genes and Genomes (a database resource for understanding high-level functions and utilities of the biological system)
LC: liver cirrhosis
LPS: lipopolysaccharide
MS: mass spectrometer
OTUs: operational taxonomic units
PCA: principle component analysis
PCoA: principal coordinate analysis
PICRUSt: phylogenetic investigation of communities by reconstruction of unobserved states [a bioinformatics software package designed to predict metagenome functional content from marker gene (e.g. 16s rRNA) surveys and full genomes]
PAMPs: pathogen-associated molecular patterns
PT.A: prothrombin activity
PT: prothrombin time
SCFA: short-chain fatty acid
TBA: total bile acid
TBIL: total bilirubin
UPLC: ultra performance liquid chromatography
