# Supplementary material for: Splenectomy Leads to Amelioration of Altered Gut Microbiota and Metabolome in Liver Cirrhosis Patients
Source: Front Microbiol. 2018 May 15;9:963. doi: 10.3389/fmicb.2018.00963 (PMC5962811; doi:10.3389/fmicb.2018.00963)
Supplement: Supplementary file 5 [file Presentation_1.pdf]

DNA extraction and PCR amplification

The final DNA concentration and purification were determined by NanoDrop 2000 UV-vis spectrophotometer (Thermo Scientific, Wilmington, USA), and DNA quality was checked by 1% agarose gel electrophoresis. The PCR reactions were conducted using the following program: 3 min of denaturation at 95 °C, 27 cycles of 30 s at 95 °C, 30s for annealing at 55 °C, and 45s for elongation at 72 °C, and a final extension at 72 °C for 10 min. PCR reactions were performed in triplicate 20 μL mixture containing 4 μL of 5 × FastPfu Buffer, 2 μL of 2.5 mM dNTPs, 0.8 μL of each primer (5 μM), 0.4 μL of FastPfu Polymerase and 10 ng of template DNA. The resulted PCR products were extracted from a 2% agarose gel and further purified using the AxyPrep DNA Gel Extraction Kit (Axygen Biosciences, Union City, CA, USA) and quantified using QuantiFluor™ -ST (Promega, USA) according to the manufacturer's protocol

Processing of sequencing data

Raw fastq files were demultiplexed, quality-filtered by Trimmomatic and merged by FLASH with the following criteria: (i) The reads were truncated at any site receiving an average quality score <20 over a 50 bp sliding window. (ii) Primers were exactly matched allowing 2 nucleotide mismatching, and reads containing ambiguous bases were removed. (iii) Sequences whose overlap longer than 10 bp were merged according to their overlap sequence.

UPLC-MS

Faecal water was extracted by taking a weighed sample of thawed stool, and mixing with methanol in a ratio of 3 ml/g. 20 μL of internal standard (0.3 mg/ml 2-Chloro-D-phenylalanine) was then added. And all samples were grinded, then ultrasonic extraction in the ice water for 30 min, and centrifuged at 10000 rpm, 4 °C for 10 min. 200 μL of supernatant was transferred to vial for UPLC-MS analysis.

LC Conditions:

Column: Acquity BEH C18 column (100 mm × 2.1 mm i.d., 1.7 μm; Waters, Milford, USA). Solvent: The column was maintained at 45 °C and separation was achieved using the following gradient: 5%B–80% B over 0–10 min, 80%B–100% B over 10.0–12.5 min, 100%B–5%B over 12.5–12.6 min, and 12.6–14.0 min holding at 5 % B at a flow rate of 0.40 mL/min, where B is acetonitrile (0.1% (v/v) formic acid) and A is aqueous formic acid (0.1% (v/v) formic acid) . Injection Volume was 3.00 μL and Column Temperature was set at 45.0 °C.

The source temperature and desolvation temperature was set at 120°C and 500°C, respectively, Nitrogen was used as both the desolvation gas (900 L/h) and cone gas (50 L/h).

Centroid data was collected from 50 to 1,000 m/z with a scan time of 0.1 s and interscan delay of 0.02 s over a 13 min analysis time using the following parameters. The parameters used were retention time(RT) range 0.5–14.0 min, mass range 50–1,000 Da, mass tolerance 0.01 Da. Isotopic peaks were excluded for analysis, noise elimination level was set at 10.00, minimum intensity was set to 15 % of base peak intensity and, finally, RT tolerance was set at 0.01 min. The Excel file was obtained with three dimension data sets including m/z, peak RT and peak intensities, and RT–m/z pairs were used as the identifier for each ion. The resulting matrix was further reduced by removing any peaks with missing value (ion intensity = 0) in more than 60 % samples. The internal standard was used for data QC (reproducibility).

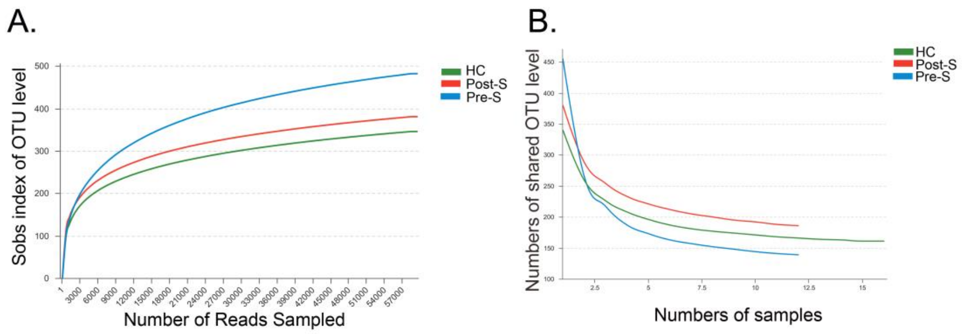

Figure S1. (A) Rarefaction curves exhibited the OTU richness in Pre-S and Post-S group compared with HC. (B) Core OTU altered with the sample's amount in Pre-S and Post-S group compared with HC.

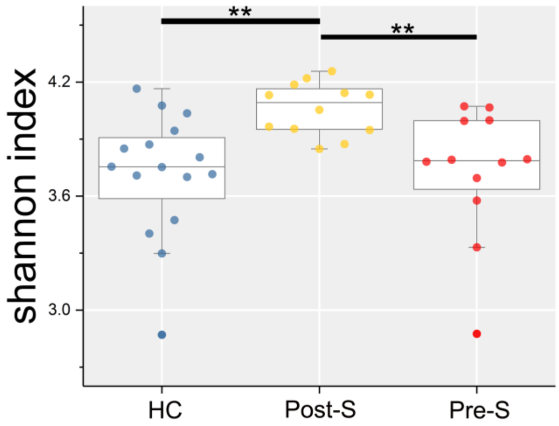

Figure S2. The Shannon indices were used to estimate the richness diversity of gut microbiota. Wilcoxon rank-sum test , **p<0.01

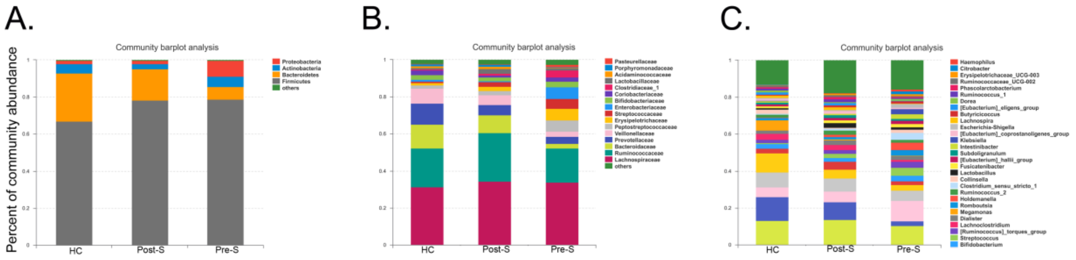

Figure S3. The relative abundance of faecal bacterial phyla (A), family (B) and genus (C) were clustered into each groups. All OTUs with lower abundances were grouped as "others".

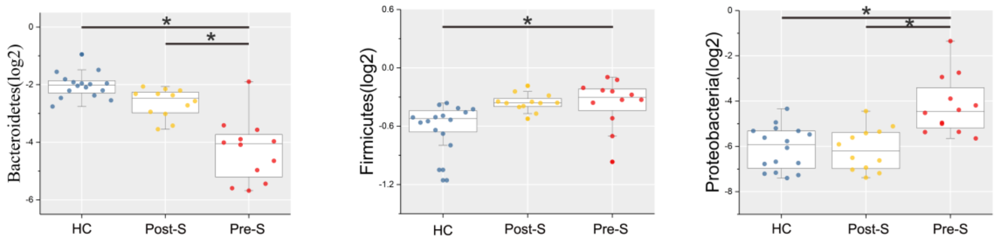

Figure S4. The relative abundance of *Firmicutes*, *Bacteroidetes* and *Proteobacteria* were calculated by log2, *p<0.05. Boxes represent the 25th–75th percentile of the distribution; the median is shown as a thick line in the middle of the box; whiskers extend to values with 1.5 times the difference between the 25th and 75th percentiles.

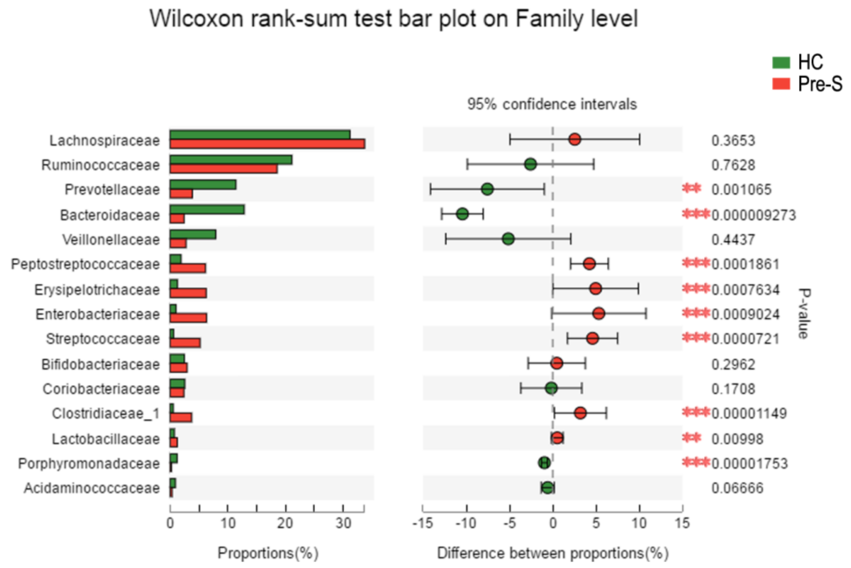

Figure S5. Comparisons of the relative abundance at the family levels in Pre-S and HC; *p< 0.05; **p< 0.01; ***p< 0.001.

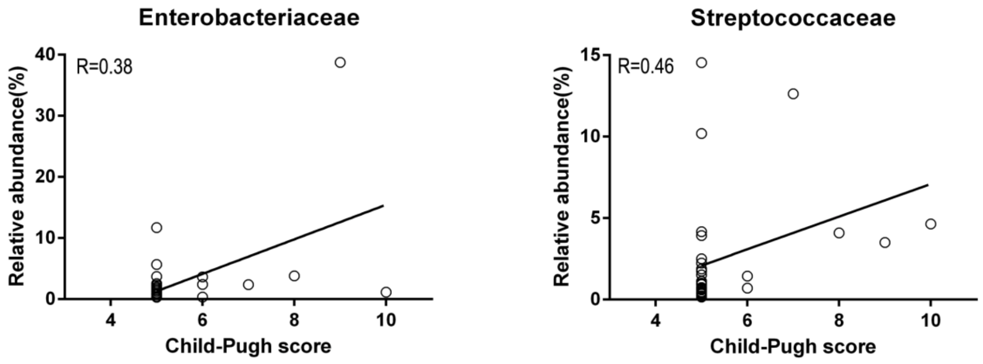

Figure S6. Correlation between Child-Pugh score and relative abundance of *Enterobacteriaceae* and *Streptococcaceae*. Spearman rank correlation, $p<0.05$.

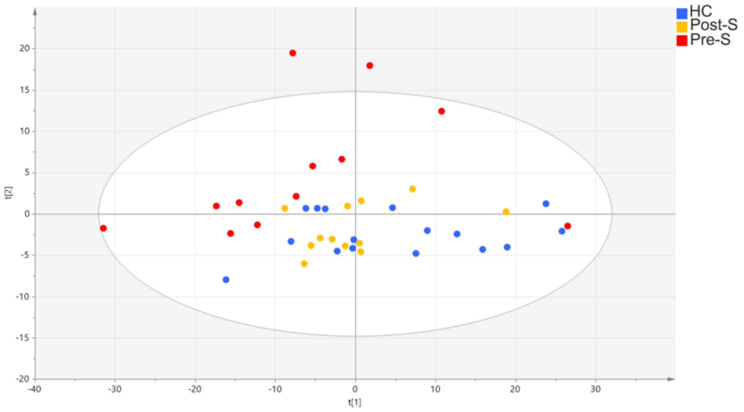

Figure S7. Principle component analysis (PCA) of KEGG pathways (levelⅢ) of HC, Post-S and Pre-S.

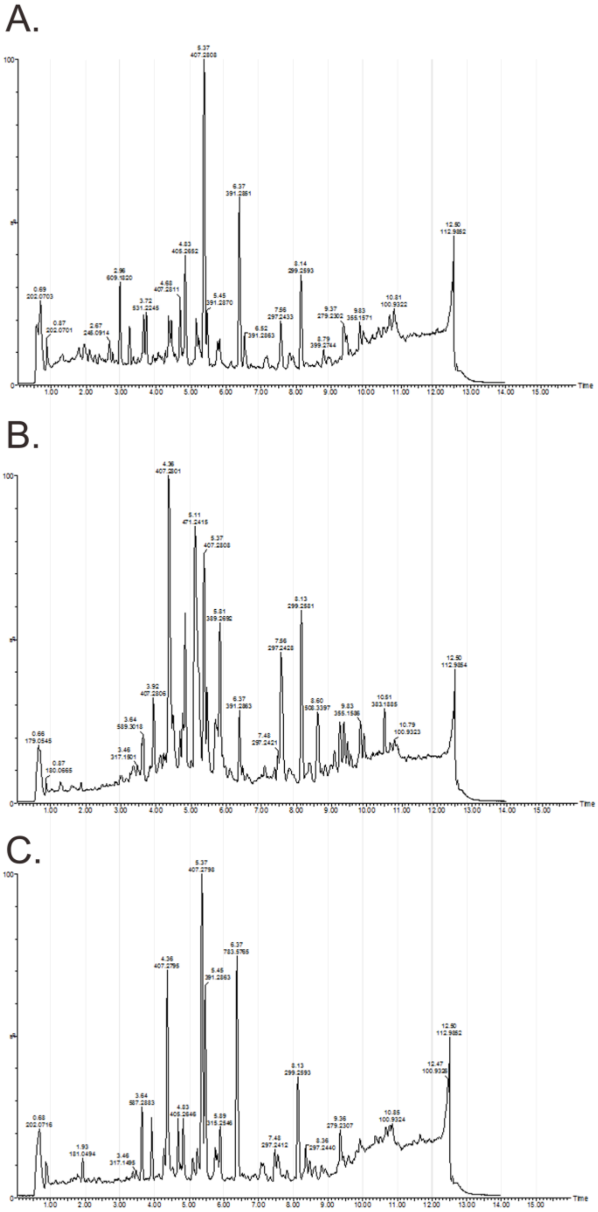

Figure S8. Representative LC/MS total ion chromatograms of the fecal samples from (A) HC, (B) Pre-S, and (C) Post-S.

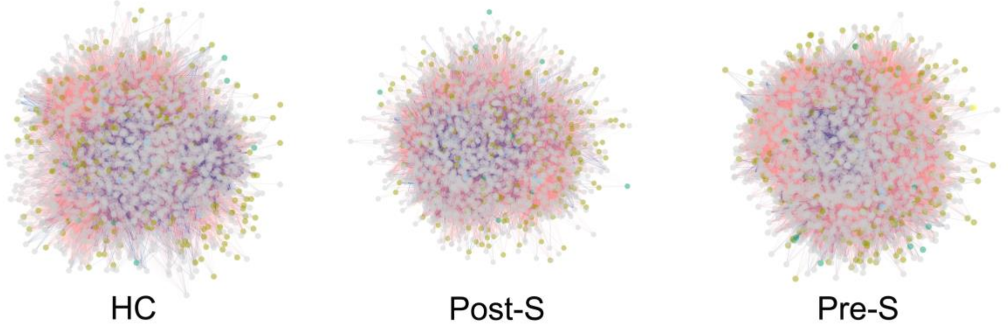

Figure S9. Correlation network analysis between all genera, metabolites and clinical parameters. Legend common for figures HC, Post-S, Pre-S: The complex correlation network represented parameters that were linked with a correlation coefficient>0.6 (negative or positive) and with a p value<0.05. Yellow nodes represent bacterial taxas, grey ones represent the serum metabolites while green ones represent indicate clinical parameters. Red edges represented positive correlation between connected nodes and blue edges indicated negative correlations.

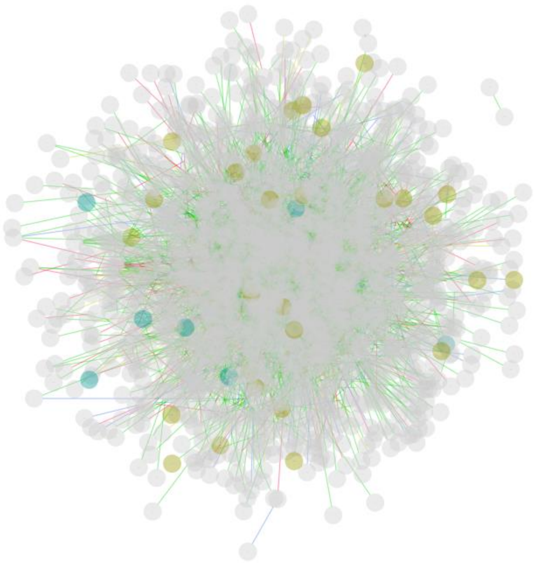

Figure S10. Correlation difference before and after splenectomy in patients . This figure shows the correlations that significantly changed between the before and after splenectomy state(p<0.05); While the color coding of the nodes is similar to figure S9. Blue edges demonstrate linkages that were positive in the Pre-S goup but became negative in Post-S; Red edges represent correlations that changed from negative to positive; Green and yellow edge represent correlations that keep positive and negative respectively but with significant change (Student t test, p<0.05).

Table S1. Perioperative data of patients

Patient number	n=12
Operative approach	
Laparoscopic Splenectomy	5(42)
Open Splenectomy	7(58)
Operation time, min	186.3±90.6（range，128.7-243.8）
Estimated blood loss, ml	970.8±897.1（range，400.9-1540.8）
In-hospital stay, d	18.3±4.1（range，15.7-20.9）
Complication	
Portal vein thrombosis	3(25)
Infectious complication	4(33)
Postoperative hemorrhage	1(8)
Ascites	1(8)
Pancreatic fistula	0(0)
Liver failure	0(0)
Mortality	0(0)
Reoperation	1(8)

Values are expressed as the means±SD, numbers in parenthesis are %.

Table S2. Comparison of network topology Pre-S, Post-S and HC.

	**HC**	**Post-S**	**Pre-S**
Number of nodes	2248	2245	2242
Isolated nodes	0	0	0
Connected components	1	1	1
Average number of neighbors	71.646	75.721	82.384
Network density	0.032	0.034	0.037
Clustering coefficient	0.439	0.468	0.469
Network radius	3	3	3
Characteristic path length	2.481	2.470	2.376
Network centralization	0.189	0.204	0.195
Shortest path	5051256	5037780	5024322
Network heterogeneity	1.119	1.162	1.036

Intersection indicates the nodes and network common to Pre-S, Post-S and HC. The table shows that the majority of nodes involved were common (intersection) between the groups while the network density (average number of neighbors and network density) changed after splenectomy.
